# Report on the Prevalence of Disease in Chicago, for the Months of April, May and June, 1867

**Published:** 1867-08

**Authors:** N. S. Davis

**Affiliations:** Prof. Practical and Clinical Medicine, Chicago Medical College


ARTICLE XXXVI.

### REPORT ON THE PREVALENCE OF DISEASE IN CHICAGO, FOR THE MONTHS OF APRIL, MAY, AND JUNE, 1867.

By N. S. DAVIS, M.D., Prof. Practical and Clinical Medicine, Chicago Medical College.

Read to the Chicago Medical Society.

Since my previous sanitary report, ending March 31st, 1867, nothing of special interest has occurred in relation to the prevalence of diseases in this city. The predominant meteorological characteristics of April were cold and dry, and those of May, cold and wet. During the latter month, the rains were so frequent and copious that the surface of our city plot was constantly saturated and sometimes covered with fresh water. During both of the months named, the prevailing temperature was so much below the average for a series of years that the progress of vegetation was two weeks later than usual. With the exception of some severe cases of typhoid pneumonia, the diseases prevalent during these two months were chiefly catarrhal and rheumatic, giving rise to a low ratio of mortality.

The first seven days of June were warm, sultry, with wind constantly from the south and south-west, and frequent showers of rain. On the 8th, the wind suddenly changed to the north, bringing a cold atmosphere filled with mist or fog. The 9th and 10th were clear, dry, and warm, with very light wind from the south. The 11th was warm, sultry, with copious showers from the south-west. The 12th, 13th, and 14th were clear, very warm, with light wind from the south and south-west. On the night of the 14th, the wind changed to the north-east, and remained there during the 15th and 16th, bringing a cool atmosphere, with mist and fog. From the 17th to the 22d, the mornings were almost uniformly warm and clear, with south or south-west wind, and the evenings and nights cool, bracing, with wind from the north-west and north. The 22d was ushered in with a hot, sultry atmosphere and south-west wind.

Thunder showers followed in the afternoon, with a change of the wind to the north-west, and much cooler. The evening was cool, with a brilliant display of atmospheric electricity. From the 23d to the 28th, the mornings were clear, hot, and dry, with only slight rain on the 27th. The wind was in the south or south-west every morning, and in the north-west or north, with a cool atmosphere, in the evening. The 29th and 30th were clear, dry, and very warm, with south winds.

In regard to local sanitary conditions, it may be said that abundant materials, in the form of stable manure, contents of outside privies, and refuse matters, existed throughout the city for engendering the products of animal and vegetable decomposition. But the low temperature and abundance of fresh-fallen water during the months of April and May prevented any deleterious action on such materials. Although the temperature of June was much higher, with less rain, yet, with not more than three exceptions, the nights were cool and bracing; and the dry days were no more than sufficient to expel the excess of water that had accumulated upon the surface the previous month. Hence, it may be said that both atmospheric and local causes were favorable to the preservation of health during the three months under consideration. And this is in consonance with the actual mortality, as shown by the Health-Officer's reports.

The gross mortality in the city was, for April, 278; May, 241; June, 283. In 1866, it was, for April, 278; May, 275; June, 319; showing a decrease of 71 during the three months of 1867, as compared with the corresponding months of 1866. This decrease consists almost exclusively in the smaller number of deaths from bowel-affections in the months of May and June.

Although the month of June, 1867, gave a low ratio of mortality in the aggregate, and a smaller number of deaths from bowel-affections than the same month in 1866, yet the relations between certain atmospheric conditions and attacks of the latter class of diseases was clearly traceable. As already stated, the first seven days of the month were warm, *showery*, and damp, with prevalence of south winds; conditions which are generally accompanied by deficiency of both ozone and free electricity. It was during the last two of the seven days, that I saw the first cases of cholera-infantum and the two first cases of cholera-morbus in adults the present season. I saw no new cases until the 12th, which was very warm with south wind, and the preceding day had been equally hot, with showers of rain. On the morning of the 16th, I was called to a woman, aged about 55 years, at 448 State Street, presenting all the symptoms of severe spasmodic cholera. She had been taken suddenly, between 1 and 2 o'clock A.M. The vomiting and purging had been frequent, copious, and rice-water in appearance; skin corrugated, cool; eyes sunken; voice very husky; pulse small and feeble; cramps in the muscles of the extremities severe and frequent; and at the time of my visit (7 o'clock A.M.) the discharges from the bowels were involuntary. The patient, however, recovered under diligent treatment. This patient slept in a small room adjoining the kitchen, without any window, and exceedingly deficient in ventilation. No other well-marked cases either of cholera-morbus or of serous diarrhœa came under my observation until the 22d.

From that time, cases of ordinary diarrhœa and cholera-morbus in infants were observed almost every day until the end of the month. But the mortality in this class of cases was small.

While the Board of Health has been acting diligently since its appointment, there remain many streets, alleys, and lots, from which the surface water has found no exit except by evaporation: and hundreds of full out-door privies and manure-heaps exist, more especially in Wards 6, 7, 11, 12, 14, and 16. Up to the end of June, enough dry weather had occurred simply to dissipate the excess of fresh water that had accumulated during the month of May; and, hence, but little influence has been everted thus far by emanations from the soil proper.

If the month of July should be hot and dry, it will develop the full influence of those miasms resulting from the action of high temperature on the local accumulations in our surface soil in the process of dessication; and will, consequently, determine the character of diseases for the present summer.

